# Action mechanism of corticosteroids to aggravate Guillain-Barré syndrome

**DOI:** 10.1038/srep13931

**Published:** 2015-09-10

**Authors:** Yu-Zhong Wang, Hui Lv, Qi-Guang Shi, Xu-Tao Fan, Lei Li, Anna Hiu Yi Wong, Yan-Lei Hao, Chuan-Ping Si, Cui-Lan Li, Nobuhiro Yuki

**Affiliations:** 1 Department of Neurology, Affiliated Hospital of Jining Medical College, Jining, Shandong Province, 272000, People’s Republic of China; 2 Central Laboratory, Affiliated Hospital of Jining Medical College, Jining, Shandong Province, 272000, People’s Republic of China; 3 Graduate School of Tianjin Medical University, Tianjin, 300071, People’s Republic of China; 4 Department of Spine Surgery, Affiliated Hospital of Jining Medical College, Jining, Shandong Province, 272000, People’s Republic of China; 5 Department of Pathology, Affiliated Hospital of Jining Medical College, Jining, Shandong Province, 272000, People’s Republic of China; 6 Department of Medicine, Yong Loo Lin School of Medicine, National University of Singapore, 117599, Singapore; 7 Department of Immunology, Jining Medical College, Jining, Shandong Province, 272067, People’s Republic of China; 8 Department of Physiology, Yong Loo Lin School of Medicine, National University of Singapore, 117599, Singapore; 9 Brain and Mind Centre, University of Sydney, 94-100 Mallett St, Camperdown NSW 2050, Australia

## Abstract

Corticosteroids have been proved to be ineffective for Guillain-Barré syndrome, but the mechanism remains unknown. In a rabbit model of axonal Guillain-Barré syndrome, treatment with corticosteroids significantly reduced macrophage infiltration in the spinal ventral roots and the survival rate as well as clinical improvement. On 30^th^ day after onset, there was significantly higher frequency of axonal degeneration in the corticosteroids-treated rabbits than saline-treated rabbits. Corticosteroids may reduce the scavengers that play a crucial role for nerve regeneration, thus delay the recovery of this disease.

Guillain-Barré syndrome (GBS) is the most frequent cause of acute flaccid paralysis worldwide. Clinical trials have demonstrated that corticosteroids treatment cannot benefit the recovery of patients with GBS^1^,^2^. However, its action mechanism remains unknown. GBS is divided into demyelinating and axonal subtypes, namely acute inflammatory demyelinating polyneuropathy (AIDP) and acute motor axonal neuropathy (AMAN)^3^,^4^. Binding of the autoantibodies to peripheral nerves may activate complement *in situ*, resulting in the nerve damage in both AIDP and AMAN. In AIDP, macrophage infiltration occurs after complement-mediated damage^3^. In a rabbit model of AMAN, macrophage infiltration occurs at the early recovery phase but not at the acute progressive phase of disease^5^. These findings suggest that complement plays a crucial role for the nerve injury, and that macrophages are scavengers for the injured nerve fibers.

We hypothesized that corticosteroids inhibit the migration of macrophage into the injured peripheral nerve and delay the recovery of GBS. In this study, we explored the effect of methylprednisolone on macrophage infiltration and clinical improvement of AMAN rabbits.

## Results

There was no significant difference in any of the baseline characteristics between two groups of rabbits: inoculation times, days from first inoculation to onset, clinical score at onset and body weight (Table 1).

**Figure 1:**
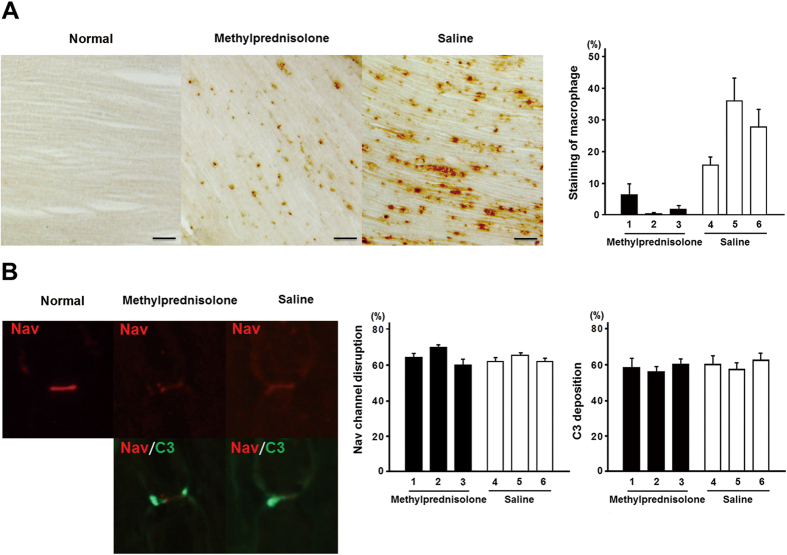
(**A**) Staining of macrophage infiltration in the spinal ventral roots of acute motor axonal neuropathy (AMAN) rabbits one week after disease onset in methylprednisolone and saline groups. Scale bars indicate 200  μm. There was significant reduction of macrophage infiltration in methylprednisolone group than saline group. The numbers 1–6 represent the serial number of AMAN rabbits in different groups. The results were shown as mean ± standard error. (**B**) Voltage-gated sodium (Nav) channels cluster disruption and C3 deposition at the nodes of Ranvier in spinal ventral roots of AMAN rabbits. Representative immunofluorescence images of longitudinal sections of spinal ventral roots from the rabbits (50  μm). The activated C3 fragments were stained in green, Nav channels in red. As shown, the nodal Nav channel cluster is markedly disrupted together with the activated C3 fragment deposition in both methylprednisolone group and saline group. There was no difference in frequency of both Nav channel cluster disruption and activated C3 fragment deposition between methylprednisolone and saline groups. The numbers 1–6 represent the serial number of AMAN rabbits in different groups. The results were shown as mean ± standard error.

In experiment 1, macrophage infiltration was significantly decreased in the methylprednisolone group than the saline group (Fig. 1A); whereas, there was no difference in the frequency of both Nav channel cluster disruption and activated C3 fragment deposition between the two groups (Fig. 1B).

**Figure 2:**
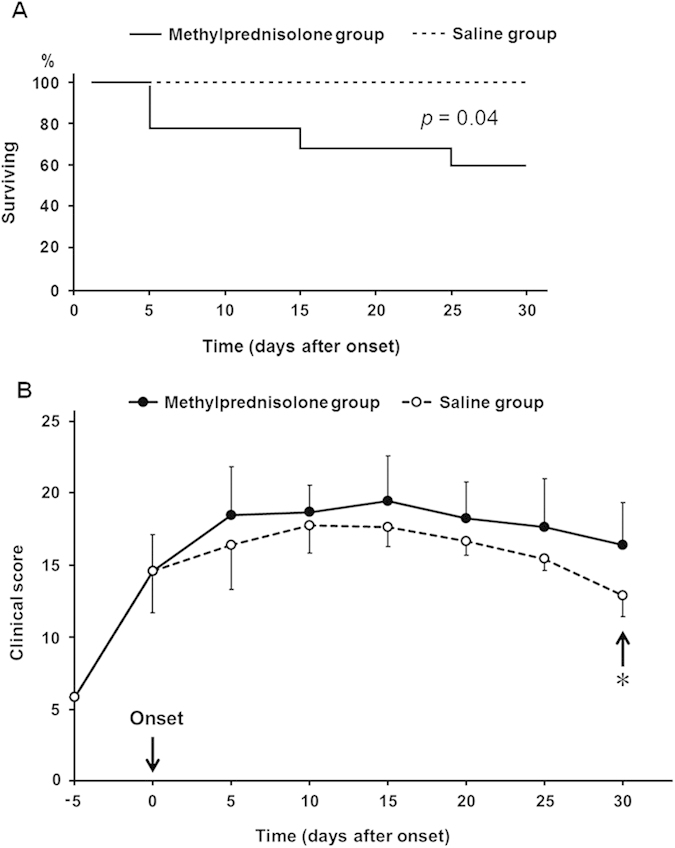
(**A**) Survival curves of rabbits up till 30 days after disease onset were shown. Five out of twelve rabbits died by day 30 after disease onset in methylprednisolone group. There was significant difference between the survival curves of methylprednisolone and saline groups (*p* = 0.04). (**B**) Changes in the clinical score (mean ± standard error) during the 30 days after disease onset. *p = 0.01.

**Figure 3:**
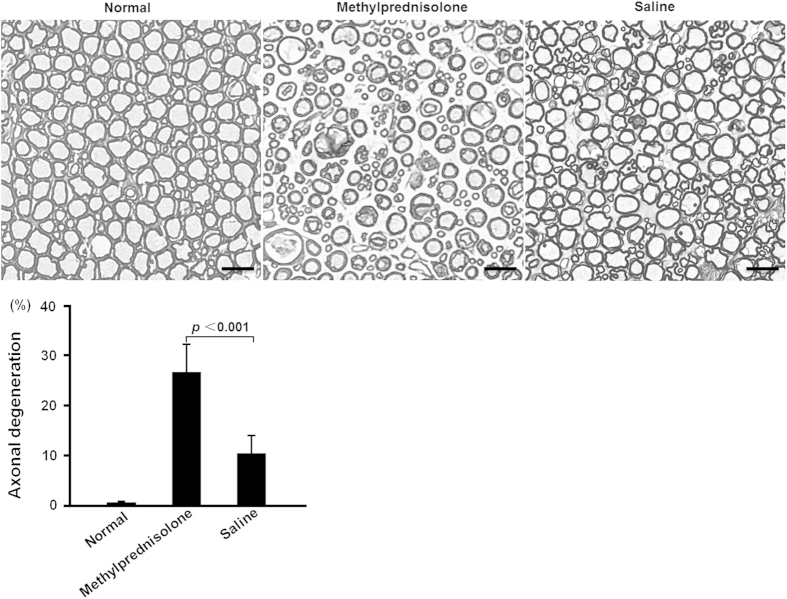
Histological changes in the ventral roots of AMAN rabbits and the normal control were shown. Scale bars indicate 50 μm. The frequency of axonal degeneration was significantly higher in methylprednisolone group (n = 7) than the saline group (n = 9) on the 30^th^ day after the initiation of treatment (*p* < 0.001). The results were shown as mean ± standard error.

In experiment 2, three AMAN rabbits in the saline group were excluded because of the unexpected injury and the others survived until the end point. In the methylprednisolone group, five out of 12 rabbits (42%) died within 30 days after disease onset. After the initiation of methylprednisolone treatment, three rabbits died on the 5^th^ day, one died on the 15^th^ day and one died on the 25^th^ day. The autopsy findings showed gastrointestinal haemorrhage in two of the rabbits who died on the 5^th^ day. An obvious cause of death in the other rabbits could not be found. The life-table method showed significantly fewer survival in the methylprednisolone group than the saline group (*p* = 0.04) (Fig. 2A). On the 30^th^ day after disease onset, the mean clinical scores were significantly lower in the saline group than the methylprednisolone group (*p* = 0.01) (Fig. 2B). The frequency of axonal degeneration was significantly higher in methylprednisolone group (n = 7) than saline group (n = 9) (*p* < 0.001) (Fig. 3).

## Discussion

Corticosteroids are the most commonly used drugs worldwide for autoimmune diseases because of its cost effectiveness. However, the application of corticosteroids in the treatment of GBS remains to be disappointing for long time. Observational studies have demonstrated that steroids treatment has no beneficial effect on GBS during the past decades^1^,^2^, but no studies have elucidated the exact mechanism so far. In the current study, we presented the first evidence that steroids reduce the macrophage infiltration and thus delay the regeneration of injured nerves in a rabbit GBS model.

Anti-GM1 and anti-GD1a IgG antibodies cause complement-mediated disruption of Nav channel clusters at the nodes of Ranvier in the rabbit and mouse models of AMAN^5^,^6^. In this study, methylprednisolone reduced neither the C3 deposition nor the disruption of Nav channels, suggesting that corticosteroids do not reduce the complement-mediated nerve injury in the AMAN rabbits. In contrast, we found significant reduction of macrophages infiltration in the ventral roots of methylprednisolone-treated AMAN rabbits, which confirms our hypothesis that corticosteroids inhibit the migration of macrophage into the peripheral nerve.

Macrophages engulf and digest the cellular debris and foreign microbes, being divided into a killing/inhibitory type (M1 macrophage) and a heal/growth promoting type (M2 macrophage)^7^. During Wallerian-like degeneration in the peripheral nerves, a model for studying the cellular response to remove debris of myelin and axons by non-immune mechanisms, macrophages are recruited specifically to degenerating fibers without the presence of T cells. Recruitment of large numbers of macrophages did not occur until the fiber degeneration is underway^8^. In AIDP, at the stage of complement deposition and early myelin vesiculation, macrophages were rarely associated with fibers. However, at later times, when myelin disruption was more advanced, macrophages were abundantly recruited^3^. In a rabbit model of AMAN, macrophage invasion was significantly more frequent at the early recovery phase than the acute progressive phase^5^. These findings supported that in GBS, infiltrated macrophages are scavenger to remove the debris of myelin and axon in injured nerve fibers. It has been demonstrated that high concentration of steroids exerts immunosuppressive effects on macrophages^9^ and inhibits the accumulation of macrophages into the injury site^10^. In this study, we observed significantly less macrophage infiltration and higher frequency of axonal degeneration in AMAN rabbits treated with methylprednisolone. Our results suggest that in AMAN rabbits, methylprednisolone reduces the clearance of injured axons by macrophages and thus delay the axonal regeneration.

Previous clinical trials showed that a short course of high-dose steroids given alone in early stage of GBS was ineffective^1^,^2^. To further confirm the effect of corticosteroids in the AMAN model, we compared the difference in both survival and clinical scores between methylprednisolone and saline groups. As expected, methylprednisolone did not promote the recovery of AMAN rabbits. In contrast, it increased the mortality and reduced the clinical improvement in the methylprednisolone group, which was consistent with that there was less improvement of disability grade in clinical trials^1^,^11^,^12^. Corticosteroids treatment was found to have complications in patients with GBS^2^,^11^,^13^,^14^. The autopsy results showed that methylprednisolone treatment produced gastrointestinal haemorrhage in AMAN rabbits. These adverse events together with the continuously poor conditions caused by delay of the axonal regeneration may well explain the mortality and delayed recovery of AMAN rabbits in methylprednisolone group.

In conclusion, corticosteroids inhibit the recruitment of scavengers, which are helpful for the nerve regeneration, resulting in the delay of clinical improvement in GBS.

## Methods

### Induction of AMAN model

AMAN rabbits were produced as previously described^15^. Clinical scale of the rabbits was observer-blinded monitored daily as described previously^16^. Disease onset was defined as a clinical score of 10 points or more. At disease onset, rabbits were divided randomly into methylprednisolone group and saline one. The experiments were approved by the Institutional Animal Care and Use Committee of the Affiliated Hospital of Jining Medical College and performed in accordance with the United States Public Health Service’s Policy on Use of Laboratory Animals.

### Experiment 1

In methylprednisolone group (n = 3), methylprednisolone was injected into the AMAN rabbits through the ear vein at a dose of 7 mg/kg per day for a total of five days. The dosage of methylprednisolone was calculated according to the dose used in previous clinical trials^2^. For the saline group (n = 3), same volume of normal saline were injected into the rabbits per day for a total of five days. On the 7^th^ day, the rabbits were perfused transcardially and the ventral roots of their lumbar cords were prepared as described elsewhere^5^.

The immunohistochemistry was performed as previously reported^5^. For the staining of Nav channels cluster and activated C3 fragment deposition, the 6-μm-thick cryosections of ventral roots from the AMAN rabbits were incubated with mouse anti-Nav channel IgG antibodies (Sigma) and fluorescein isothianate-conjugated anti-rabbit C3c antibodies (Nordic Immunological Laboratory, Tilburg, The Netherlands) first, then with Alexa Flour 568-conjugated anti-mouse IgG antibodies (Invitrogen, Carlsbad, CA). For the immunostaining of macrophage, the 50-μm-thick cryosections were incubated with mouse anti-rabbit macrophage IgG antibodies (clone, RAM11) (Dako Cytomation, Carpinteria, CA) first and then the reaction were visualized using peroxidase-conjugated SABC kit (ready to use) for mouse IgG (Boster, Wuhan, China). All of the images were captured using Axio Observer A1 inverted fluorescence microscope (Zeiss, Jena, Germany).

Numbers of disrupted Nav channel clusters and C3 depositions were counted in two different roots for each rabbit in at least 35 microscopic fields. Average optical density of staining area of macrophage infiltration per field was measured for at least 40 images from each AMAN rabbit using Image-pro plus (version 6.0) (Media Cybernetics, Bethesda, MD). The quantification was observer-blinded. The results were shown as mean ± standard error. Mann-Whitney *U* test was used to compare the frequency of disrupted Nav channel clusters and C3 depositions and the macrophage infiltration between different groups.

### Experiment 2

In both groups (methylprednisolone group, n = 12; saline group, n = 12), AMAN rabbits were monitored daily until 30 days after the initiation of treatment. After the follow-up period, the rabbits were perfused transcardially and the ventral roots of their lumbar cords were excised for the toluidine blue staining as previously described^17^. The number of degenerative axons from every four frame area (single frame, 0.03 mm^2^) in the ventral roots of the lumbar cord was counted by a blinded observer^17^. The frequency of degenerative axons (the ratio of the number of degenerative axons to the total number of axons of all frame areas) was calculated for each rabbit. The results were shown as mean ± standard error. Mann-Whitney *U* test was used to compare the frequency of degenerative axons between different groups. Five normal rabbits without immunization or treatment constituted the histologic controls. Life-table method was used to evaluate the effect of methylprednisolone on AMAN rabbits surviving. A *p* value of < 0.05 was considered significant. Analysis was performed with the SPSS 19.0 analysis software by IBM (Armonk, NY).

## Additional Information

**How to cite this article**: Wang, Y.-Z. *et al.* Action mechanism of corticosteroids to aggravate Guillain-Barré syndrome. *Sci. Rep.* **5**, 13931; doi: 10.1038/srep13931 (2015).

## Figures and Tables

**Table 1:** Baseline characteristics of the acute motor axonal neuropathy rabbits.

	Experiment 1		Experiment 2	
	Methylprednisolone group	Saline group	Methylprednisolone group	Saline group
Inoculation times	4 (3–5)	4 (3–5)	4 (3–5)	4 (3–5)
Days from first inoculation to onset	102 (77–111)	97 (76–115)	99 (72–109)	104 (79–121)
Clinical score at onset	14 (10–19)	15 (10–17)	15 (10–20)	15 (10–18)
Weight at onset (kg)	2.5 (2.1–3.2)	2.7 (2.3–3.5)	2.7 (2.3–3.3)	2.6 (2.2–3.1)

Inoculation times, days from first inoculation to onset, daily clinical score at onset, and weight at onset are given as medians and ranges. There were no statistical differences (Mann-Whitney *U* test).
